# A newly synthesized flavone avoids COMT-catalyzed methylation and mitigates myocardial ischemia/reperfusion injury in H9C2 cells via JNK and P38 pathways

**DOI:** 10.22038/IJBMS.2023.74358.16149

**Published:** 2024

**Authors:** Ye Lin, Xin Yang, Yan Li, De-jian Huang, Zhi-qin Sun

**Affiliations:** 1 School of Medical and Health Engineering, Changzhou University, Changzhou 213164, P.R. China; 2 Department of Pharmacology, Yong Loo Lin School of Medicine, National University of Singapore, Singapore 117597, Singapore; 3 Food Science and Technology Program, Department of Chemistry, Faculty of Science, National University of Singapore, Singapore 117597, Singapore; 4 National University of Singapore (Suzhou) Research Institute, Suzhou, Jiangsu 215123, P.R. China; 5 Changzhou Second People’s Hospital, Changzhou 213000, P.R. China

**Keywords:** 2, 2’-azobis - (2-amidinopropane) - dihydrochloride (AAPH), Catechol-O-methyltransferase (COMT), H9C2, Luteolin, Myocardial ischemia - reperfusion

## Abstract

**Objective(s):**

Luteolin is a flavone that provides defense against myocardial ischemia/reperfusion (I/R) injury. However, this compound is subjected to methylation mediated by catechol-O-methyltransferase (COMT), thus influencing its pharmacological effect. To synthesize a new flavone from luteolin that avoids COMT-catalyzed methylation and find out the protective mechanism of LUA in myocardial I/R injury.

**Materials and Methods:**

Luteolin and 2,2’-azobis (2-amidinopropane) dihydrochloride (AAPH) were used to synthesize the new flavone known as LUAAPH-1 (LUA). Then, the myocardial ischemia/reperfusion injury cell model was established using H9c2 cells to detect the effect in myocardial ischemia/reperfusion regulation and to identify the underlying mechanism.

**Results:**

Pretreatment with LUA (20 μmol/l) substantially increased cell viability while reducing cell apoptosis rate and caspase-3 expression induced by I/R, and the protective effect of LUA on cell viability was stronger than diosmetin, which is the major methylated metabolite of luteolin. In addition, intracellular reactive oxygen species (ROS) production and calcium accumulation were both inhibited by LUA. Furthermore, we identified that LUA markedly relieved the promotive effects of I/R stimulation upon JNK and p38 phosphorylation.

**Conclusion:**

LUT pretreatment conveys significant cardioprotective effects after myocardial I/R injury, and JNK and p38 MAPK signaling pathway may be involved.

## Introduction

Over the past few decades, myocardial infarction has remained the leading cause of death in the elderly worldwide. Although advanced emergency coronary recanalization strategies such as percutaneous coronary intervention and thrombolytic therapy timely restore blood supply to the ischemic myocardium, they paradoxically induce additional structural and functional damage to the post-ischemic heart tissue, which is called ischemia-reperfusion (I/R) injury. Injuries caused by I/R not only limit the beneficial effect of reperfusion approaches but also enlarge the infarct area, thus increasing the mortality of myocardial infarction. Therefore, although the exact mechanism of I/R injury is still not well understood, it is generally recognized that excessive production of reactive oxygen species (ROS), intracellular calcium overload, inflammation, and apoptosis of cardiomyocytes are the main reasons. So far, no mature and effective intervention has been discovered for alleviating myocardial I/R injury (1), and thus, it remains a significant unmet clinical requirement.

Accumulating studies have focused on phytonutrients to provide therapeutic potential for treating myocardial I/R injury. Flavones are vast and ubiquitous in a variety of natural plants with biological benefits. Luteolin (3’,4’,5,7- tetrahydroxyflavone), as a member of natural flavone, is abundant in some medicinal plants, vegetables, and fruits such as cabbages, parsley, carrots, peppers, broccoli, apple skins, and chrysanthemum flowers (2). Emerging evidence demonstrates that luteolin exhibits a wide spectrum of pharmacological properties against oxidation, inflammation, carcinogenesis, and pathogens. It was previously reported that luteolin protected the myocardium from I/R injury *in vivo *and* in vitro* through different mechanisms (3-5). 

Despite the versatile functions of luteolin, its bioactivity may weaken when administered *in vivo*. The reason is partly attributed to the methylation of luteolin catalyzed by catechol-O-methyltransferases (COMT), which is a widespread enzyme distributed in the mammals’ organs and playing a critical role in the metabolism of catechol-containing compounds like epinephrine, norepinephrine, and l-dopa. Luteolin, containing catechol on the B ring, also serves as an excellent substrate for COMT. Several reports indicate that luteolin is methylated to diosmetin (major) and chrysoeriol (minor) by COMT (6, 7). In addition to speeding up the elimination of luteolin (8), methylation metabolites exhibit significantly lower anti-inflammatory properties compared with luteolin (9). Nevertheless, methylated derivatives of luteolin are noticeably decreased in the presence of COMT inhibitors, with more luteolin-conjugated metabolites possessing enhanced anti-inflammatory properties (9). These findings show that the anti-inflammatory activity of luteolin *in vivo* is significantly suppressed by COMT methylation. Consequently, luteolin must be protected from methylation by COMT to maintain its anti-inflammatory properties. Even though some COMT inhibitors have been developed, some of them have several drawbacks, including cell damage, insufficient bioavailability, and gastrointestinal symptoms. Therefore, safer and more efficient methods are needed to inhibit the methylation of luteolin.

Chemical structure modification is a common approach to altering the properties of compounds. In our lab, a new flavone known as LUAAPH-1 (LUA) was obtained by the reaction of luteolin and 2,2‘-azobis (2-amidinopropane) dihydrochloride (AAPH) chemically. Getting rid of the catechol hydroxyl group, this new flavone might avoid methylation by COMT when administrated *in vivo*. LUA has been proven to have strong anti-inflammatory effects in our previous research (10), and this study aimed to evaluate the protective effect of LUA on myocardial I/R injury in rat ventricular myoblasts.

## Materials and Methods

***Reagents and antibodies***

The Dulbecco’s modified Eagle’s medium (DMEM), fetal bovine serum (FBS), streptomycin, penicillin, and trypsin were purchased from Hyclone Laboratories (South Logan, UT, USA). Cell Counting Kit-8 reagent used to assess cell viability was purchased from Beyotime (Jiangsu, China). 2,7-dichlorofluorescein diacetate (DCFH-DA) applied to measure intracellular ROS production was purchased from Sigma-Aldrich (St Louis, MO, USA). Fluo-4 AM was purchased from Life Technologies (Grand Island, NY, USA). Antibodies against β-actin and GAPDH were from Santa Cruz Biotechnology (Santa Cruz, CA, USA). Other antibodies were supplied by Cell Signaling Technology (Beverly, MA, USA). All the other reagents were obtained from Sigma-Aldrich (St. Louis, MO, USA) unless otherwise stated. Luteolin was from Nanjing Plant Origin Biological Technology Co., Ltd (Nanjing, China)). AAPH was purchased from Merck & Co., Inc. (USA).

***Synthesis process of the new flavone***

**Figure 1:**
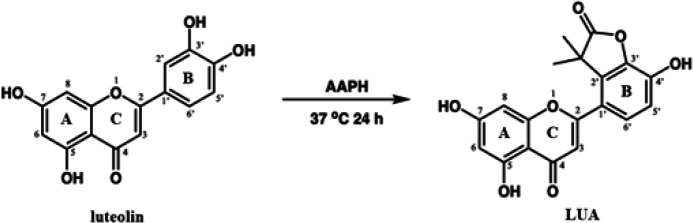
Synthetic method and molecular structure of LUA

LUA was previously synthesized and detected by our laboratory (10). In summary, we dissolved luteolin (42.9 mg, 0.15 mmol) and AAPH (244 mg, 0.9 mmol) in 75 mM phosphate buffer (pH 7.4) using a 50 ml round bottom flask. The rotary evaporator was used to stir the mixture under O_2_ at a temperature of 37 °C for 24 hr until the volatiles evaporated to dryness. The residual substance underwent purification through silica gel chromatography, with elution facilitated by EtOAc/methanol (10:1 v/v) and with an Rf of 0.35 based on TLC. Finally, we successfully obtained the anticipated product with a yield of 20.2 mg, corresponding to 47.1% of the starting material. The ESI-MS analysis of the compound was 353.07 (M-) (Figure1).

***Cell culture***

Rat ventricular myoblasts (H9c2 cells) were sourced from the American Type Culture Collection (ATCC) and cultivated in culture flasks. To support cell growth, the cells were provided with DMEM supplemented with 10% FBS and 1% penicillin/streptomycin. The cells were kept in a humidity-controlled environment with a 5% CO_2_ concentration and maintained at a temperature of 37 ℃. Cells were seeded onto culture plates at a density appropriate for each specific experiment and were cultured for at least 24 hr before experimentation.

***Simulated ischemia-reperfusion (I/R)***

**Figure 2:**
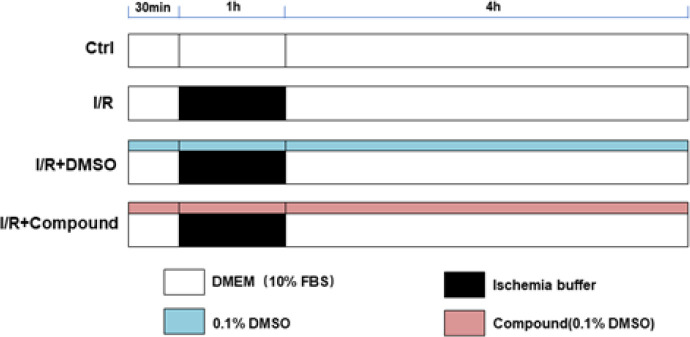
Outline of the experimental protocol to identify the cardioprotective effect of LUA on the simulated I/R injury cell model

To simulate ischemia-reperfusion that occurs *in vivo*, H9c2 cells were exposed to an ischemic buffer for 1 hr, under humidified incubation conditions with 5% CO_2_ at 37 ℃. As described previously, the ischemic buffer contained 10 mM deoxyglucose, 137 mM NaCl, 12 mM KCl, 0.49 mM MgCl_2_, 0.9 mM CaCl_2_·2H_2_O, 0.75 mM sodium dithionate, 20 mM lactate, and 4 mM Hepes, with a pH of 6.5 (11). A normal medium was transferred to the cells for four hours to trigger reperfusion. Cells were incubated either with or without the indicated compounds for 30 min before ischemia (Figure 2).

***Cell viability assay***

The viability of cells was determined using the CCK-8 colorimetric assay as directed by the manufacturer. Briefly, cells were cultivated in 96-well plates. After treatments, the supernatant was removed and replaced with 100 μl of fresh culture medium that contained 10% CCK-8 reagent. Following a 2-hr incubation period at 37 °C, the absorbance at 450 nm was measured using a Varioskan Flash microplate reader (Waltham, MA, USA). Cell viability was measured by determining the ratio of the absorbance of experimental wells to the absorbance of control wells. The absorbance ratio of experimental wells was compared to that of control wells to determine cell viability.

***Apoptosis detection with DAPI/PI staining***

The apoptosis of H9c2 cells was measured by 4’-6- diamidino-2-phenylindole (DAPI)/propidium iodide (PI) dual staining following the manufacturer’s instructions. H9c2 cells were washed three times with PBS after reperfusion. To stain the cells, they were incubated with DAPI (1 µg/ml) and PI (2 µg/ml) for 15 min at 37 °C in a dark environment. Finally, the samples were washed three times with PBS and were then visualized using a Leica fluorescence microscope manufactured by Leica Microsystems, Germany. 

***Reactive oxygen species (ROS) detection***

The 2’,7’-dichlorofluorescein diacetate (DCFH-DA) method was used to visualize intracellular ROS generation. After reperfusion, H9c2 cells in the 96-well plate were exposed to a 10 μM DCFH-DA probe and were kept for an incubation period of 30 min at 37 ℃. The fluorescence intensity was measured by exciting the cells at 485 nm and measuring their emission at 535 nm. Additionally, fluorescent images were taken using a Leica fluorescence microscope, and the fluorescence intensity of the images was accurately quantified by Image-Pro Plus.

***Measurement of Intracellular calcium***

Fluo-4 AM dye was employed to assess intracellular calcium concentrations. Fluorescence was monitored at Ex/Em 491/516 nm using a Leica fluorescence microscope. After reperfusion, H9C2 cells were washed in PBS and labeled by Fluo-4 AM probe for 30 min at 37 ℃. The fluorescent intensity of images was quantified using Image-Pro Plus.

***Western blot***

H9c2 cells were cultured in 6-well tissue culture plates. After reperfusion, the cells were washed three times in chilled PBS and lysed using RIPA lysis buffer that contained phosphatase and protease inhibitors from Thermo Fisher Scientific. The protein content of each sample was then measured using a BCA Colorimetric Protein Assay Kit sourced from the United States. Equal portions of protein from each sample were separated by using either 10 or 12% SDS-PAGE and subsequently transferred onto a PVDF membrane. To prevent non-specific binding, a solution of 5% skim milk was applied to the sample and incubated at room temperature for 1 hr. Then, the membranes were incubated overnight at 4 ℃ with the primary antibodies. The membranes underwent three washes with TBST buffer, which is a Tris-buffered saline containing 0.1% Tween 20. Following this, the membranes were incubated with suitable secondary antibodies that had been conjugated with horseradish peroxidase. This incubation period occurred at room temperature and lasted for an hour. After being washed three times with TBST, the blots were then exposed to the WesternBright ECL Detector from Advansta, USA. The intensity of the immunoreactive bands was analyzed using Image-Pro Plus and normalized to the levels of β-actin, GAPDH, or non-phosphorylated protein.

***Statistical analysis***

All results were presented as mean±SEM (n≥3). Differences were analyzed by one-way analysis of variance (ANOVA), followed by Bonferroni correction for a *post hoc* t-test using the GraphPad Prism 5 software package. A level of *P*<0.05 was considered statistically significant.

## Results

***Impact of LUA on cell viability in H9c2 cells***

**Figure 3:**
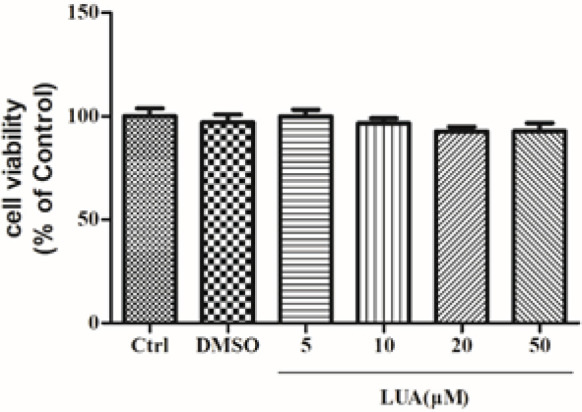
**I**mpact of LUA on cell viability in H9c2 cells

To identify the toxic effect of LUA, H9c2 cells were treated with 0.1% DMSO or different concentrations of LUA (5, 10, 20, and 50 μM) for 24 hr. As shown in Figure 3, there were no significant differences in cell viability among the groups, suggesting no toxicity of LUA on H9c2 cardiomyocytes.

***LUA protected H9c2 cells from simulated I/R***

**Figure 4:**
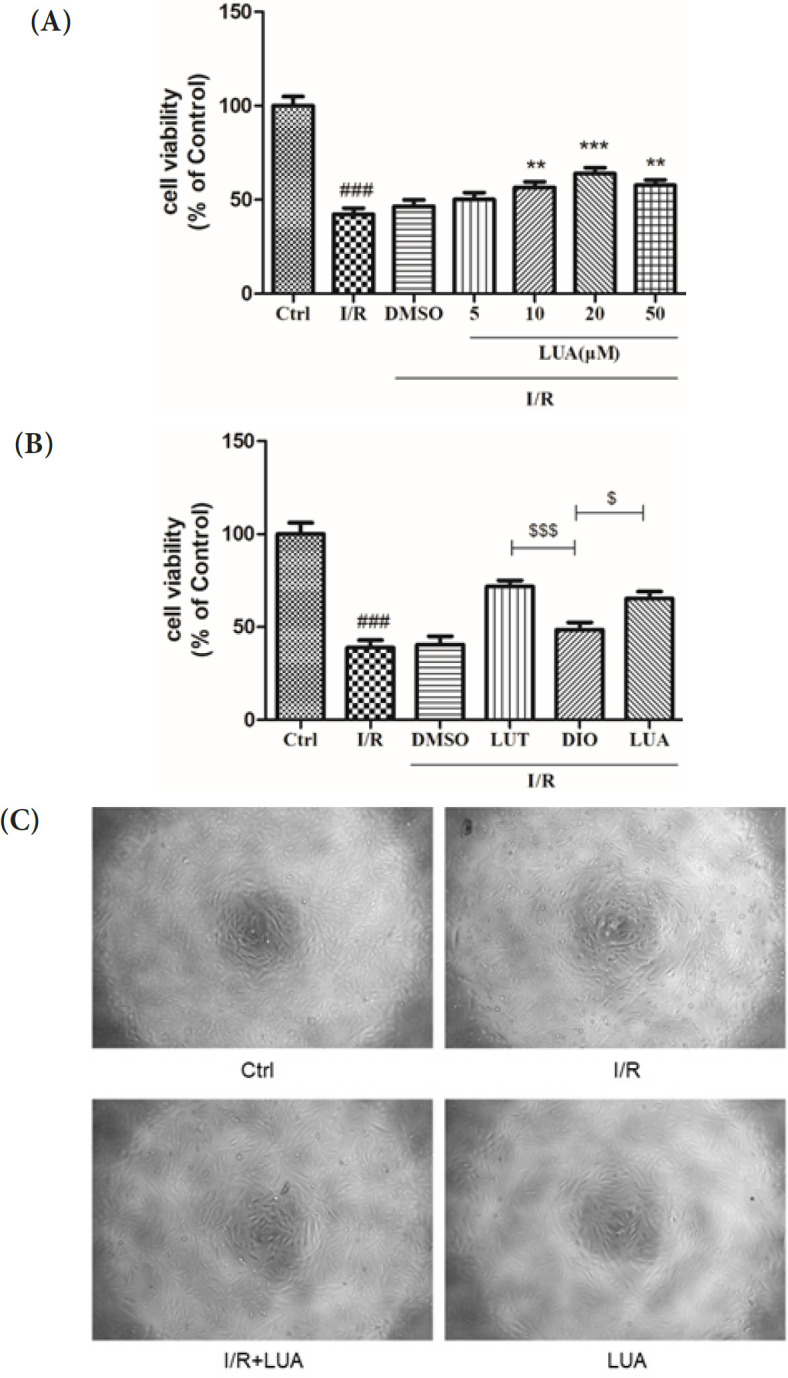
LUA protected H9c2 cells from simulated I/R

To confirm the effects of LUA on simulated I/R, we initiated by conducting tests to assess the effect of different concentrations of LUA on cell viability in H9c2 cells. As shown in Figure 4A, cell viability declined to 42.37±3.19% (*P*<0.001) after exposure to 1 hr of ischemia and 4 hr of reperfusion, whereas it increased to 56.56± 3.04% and 63.90±3.19% with 10 and 20 μM LUA respectively, indicating pretreatment with LUA protected the cells from I/R injury in a dose-dependent manner. Therefore, a dose of LUA at 20 μM was selected for further experiments. In addition, lutein, diosmetin, and LUA were compared for their protective effects on I/R injury. As shown in Figure 4B, pretreatment with diosmetin resulted in a notably weaker protective effect on cell viability compared to luteolin and LUA. In accordance with cell viability, treatment with LUA at a concentration of 20 μM also ameliorated I/R-induced morphological changes in H9c2 cells. As shown in Figure 4C, the cells in the control group maintained intact membranes and typical spindle shapes. However, cell shrinkage, rounding, and petri dish surface detachment were seen in the I/R groups and LUA pretreatment prevented the morphological changes induced by I/R.

***LUA protected H9c2 cells from simulated I/R by inhibiting ROS generation***

**Figure 5:**
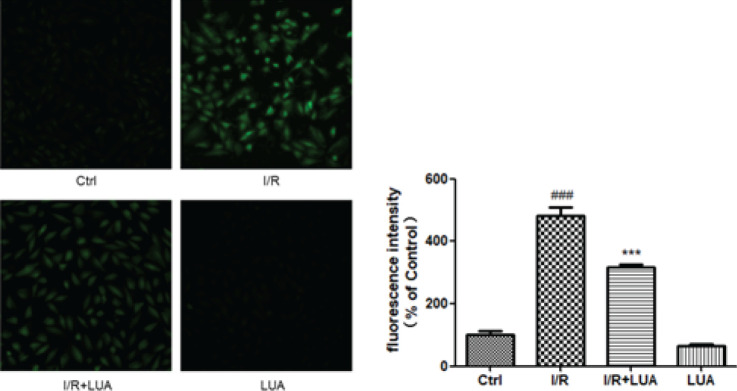
LUA protected H9c2 cells from simulated I/R by inhibiting ROS generation

The effects of LUA on ROS generation were determined using the DCFH-DA probe. As presented in Figure 5, simulated I/R sharply increased ROS accumulation in H9c2 cells compared with the control group. In comparison to the IR group, the presence of 20 μM LUA resulted in significantly reduced intracellular ROS production.

***LUA protected H9c2 cells from simulated I/R by inhibiting apoptosis***

**Figure 6:**
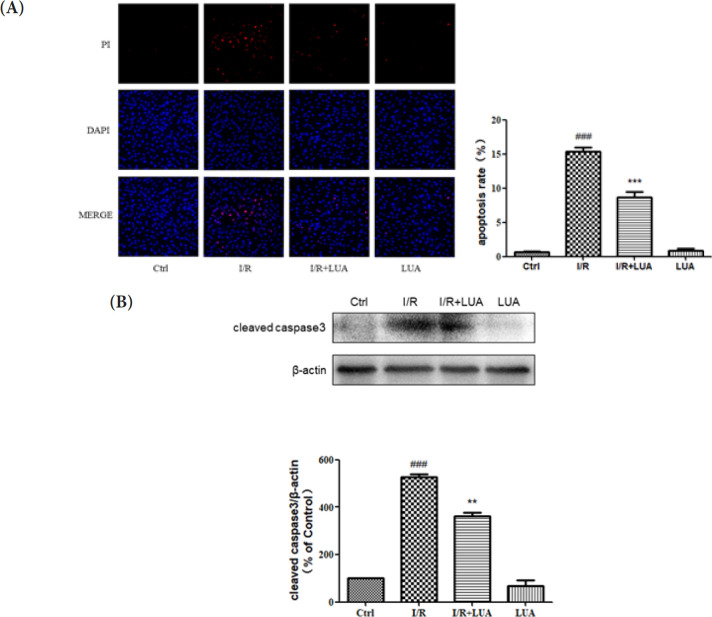
LUA protected H9c2 cells from simulated I/R by inhibiting apoptosis

To investigate the protective effects of LUA on apoptosis induced by simulated I/R in H9c2 cells, DAPI/PI staining assay was performed and cleaved Caspase-3 expression was analyzed by western blot. As shown in Figures 6A and 6B, the I/R group showed a greater apoptotic rate than the control group, accompanied by obvious cleaved Caspase-3 expression increases. However, LUA (20 μM) pretreatment significantly ameliorated these alterations caused by IR.

***LUA protected H9c2 cells from simulated I/R by mitigating intracellular calcium elevation***

**Figure 7:**
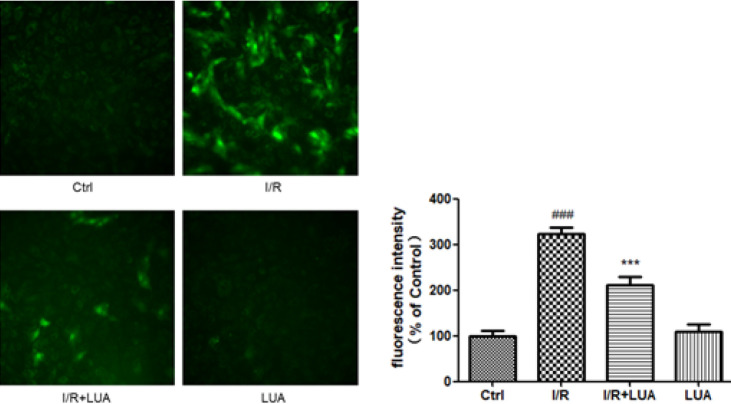
LUA protected H9c2 cells from simulated I/R by mitigating intracellular calcium elevation

To demonstrate whether LUA affected intracellular Ca^2+^ accumulation evoked by simulated I/R, the fluorescent intensity of Fluo-4 AM labeled Ca^2+^ in cells was assessed using confocal microscopy. As shown in Figure 7, the exposure of H9c2 cells to I/R caused a robust increase in Ca^2+^. However, pretreatment with LUA (20 μM) 30 min before I/R blunted the I/R-induced intracellular calcium accumulation in cardiomyocytes.

***LUA protected H9c2 cells from simulated I/R by suppressing JNK and p-38 /MAPK signal pathways***

**Figure 8:**
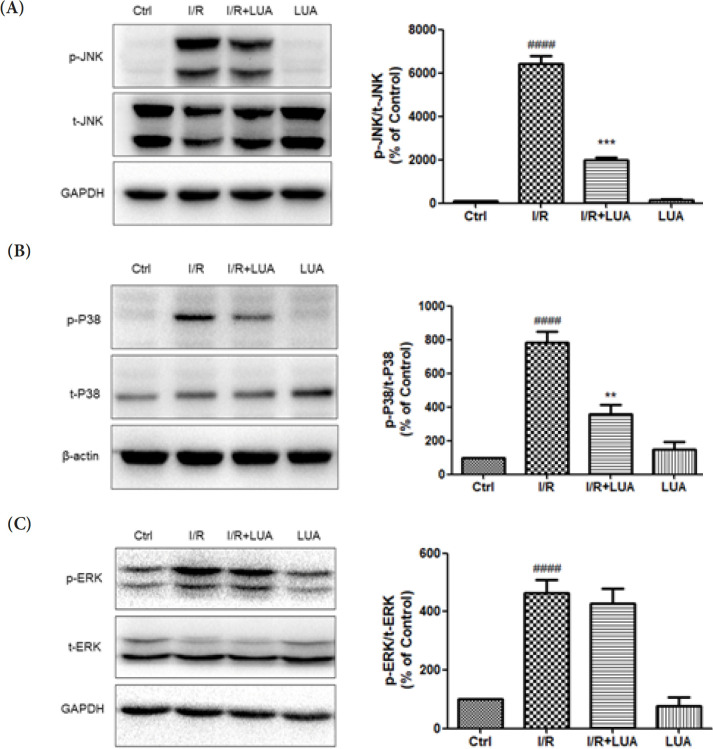
LUA protected H9c2 cells from simulated I/R by suppressing JNK and p-38 /MAPK signal pathways

To determine whether the protective effect of LUA on I/R injury in H9c2 cells was associated with the inhibition of the MAPK pathway, we detected the phosphorylation of JNK, p38, and ERK/MAPK in H9c2 cells after simulated I/R. As shown in Figure 8, a dramatic increase in the phosphorylation of JNK, p38, and ERK/MAPK was observed in the I/R group when compared to the control group. LUA (20 μM) pretreatment inhibited the I/R-induced phosphorylation of JNK and p38/MAPK but not ERK/MAPK in the I/R-stimulated cardiomyocytes.

LUA was synthesized by the chemical reaction of luteolin and AAPH at a temperature of 37 °C for 24 hr

LUA: LUAAPH-1; AAPH: 2,2’-azobis (2-amidinopropane) dihydrochloride

H9c2 cells were pretreated with vehicle (0.1% DMSO) or different compounds (LUA, LUT, and DIO) for 30 min, followed by 1 hr of ischemia and then 4 hr of reperfusion.

LUA: LUAAPH-1; LUT: luteolin); DIO: Diosmetin

H9c2 cells were exposed to either 0.1% DMSO or several concentrations of LUA (5, 10, 20, and 50 μM) for 24 hr. Cell viability was measured using the CCK-8 assay. The data were expressed as mean ± SEM (n = 6).

LUA: LUAAPH-1

(A) H9c2 cells underwent pretreatment with various concentrations of LUA for a period of 30 min before being exposed to 1 hr of ischemia and 4 hr of reperfusion. (B) Cells were then pretreated with LUT, DIO, and LUA, all at a concentration of 20 μM for 30 min before exposure to 1 hr of ischemia and 4 hr of reperfusion. Cell viability was determined by CCK-8 assay. (C) Cell morphology was observed after I/R under a phase contrast microscope (Olympus IX 51, Tokyo, Japan). The representative images were obtained from three independent experiments (40×). ###*P*<0.001 vs control group; **P<0.01, ***P<0.001 vs I/R group.

LUA: LUAAPH-1; I/R: Ischemia-reperfusion; LUT: Luteolin; DIO: Diosmetin

H9c2 cells were pretreated with LUA (20 μM) for 30 min before exposure to 1 hr of ischemia and 4 hr of reperfusion. Intracellular ROS generation was measured using DCFH-DA fluorescence (excitation= 485 nm, emission= 535 nm). ###*P*<0.001 vs control group; ***P<0.001 vs I/R group

LUA: LUAAPH-1; ROS: Reactive oxygen species; I/R: Ischemia-reperfusion

(A) Representative images of DAPI/PI staining to analyze apoptosis of H9c2 cells. (B) Cleaved Caspase-3 protein expression level was identified by western blot in H9c2 cells. ###*P*<0.001 vs control group; **P<0.01, ***P<0.001 vs I/R group

LUA: LUAAPH-1; I/R: Ischemia-reperfusion

Representative images of Fluo-4 AM staining were obtained by confocal microscopy, and the mean fluorescent intensity of each group was analyzed. ###*P*<0.001 vs control group; ***P<0.001 vs I/R group

LUA: LUAAPH-1; I/R: Ischemia-reperfusion

The total protein and phosphorylation of JNK (A), p38 (B), and ERK (C)/MAPK in the H9c2 cells were analyzed by western blot. ####*P*<0.0001 vs control group; **P<0.01, ***P<0.001 vs I/R group.

LUA: LUAAPH-1; I/R: Ischemia-reperfusion

**Figure 9:**
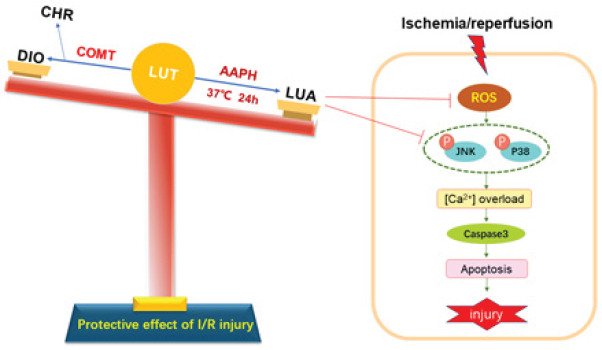
Framework of LUA synthesis and its protective effect on myocardial I/R injury

LUA: LUAAPH-1; I/R: Ischemia-reperfusion

## Discussion

The flavone subclass of flavonoids known as luteolin, which is frequently present in food plants, has a wide range of biological functions. Studies have shown that the bioavailability of luteolin is relatively low, and the reason is partly attributed to phase II metabolism such as methylation, sulfation, and glucuronidation (12). Luteolin is characterized by a C6-C3-C6 structure containing two benzene rings (called rings A and B), one oxygenated ring with a 2-3 carbon double bond (designated ring C), and hydroxyl groups on 3’,4’,5,7 carbons. Sharing a catecholic motif on the B ring; luteolin is known to be metabolized to its methylated form by COMT. Chrysoeriol and diosmetin are two methylated compounds from luteolin. Chrysoeriol is methylated at the 3’ position, while diosmetin is methylated at the 4’ position. Although two methylated metabolites were both found in the plasma and urine of rats after being administered intravenously, it was observed that COMT has a higher affinity for methylation at the 4’-hydroxyl position of luteolin compared to the 3’-hydroxyl position in human liver S9 and rat tissue (7, 13). It was reported that COMT inhibitors suppressed the methylation of luteolin, producing luteolin conjugates with more effective anti-inflammatory activities than diosmetin conjugates (9). This finding indicated that methylation of luteolin might inhibit its pharmacological effect when administrated *in vivo*.

Luteolin has been identified to possess notable free radical scavenging activities for a long time (14, 15). AAPH, an azo compound, could spontaneously produce peroxy-free radicals and has been employed as an important free radical donor. We previously discovered that luteolin scavenged free radicals supplied by AAPH and generated LUA as the predominant product (10, 16). Due to the lack of catechol structure, the new flavone may avoid COMT-mediated methylation. Our previous studies detected the anti-inflammatory effect of LUA and found it exerted a stronger anti-inflammatory effect than diosmetin in the LPS-stimulated RAW cell model (10), indicating the potentially enhanced biological activity compared with luteolin considering the enzyme metabolism. We demonstrated in this work that pretreatment with LUA significantly ameliorated simulated I/R injury in H9c2 cells, as demonstrated through an increase in cell viability, cell morphology, and reduction of apoptosis. Besides, compared to diosmetin, the primary methylated luteolin metabolite, LUA exhibited much higher protection against I/R damage. This finding implied that LUA may have a more significant effect on myocardial I/R *in vivo* than luteolin.

Convincing evidence demonstrates that reperfusion-induced oxidative stress following ischemia is the primary mechanism of myocardial I/R injury (17, 18). Some experiments have shown that luteolin has an outstanding range of means through which it thwarts oxidative damage and diminishes myocardial I/R injury, such as regulating Akt/Nrf2, Sirt1/NLRP3/NF-κB signaling pathways, increasing the activity of Superoxide dismutase (SOD) and peroxiredoxin II, as well as decreasing lipid peroxidation (4, 19-21). Studies have also revealed that diosmetin attenuated cerebral ischemia/reperfusion injury both *in vivo* and *in vitro*. This effect was achieved through the inhibition of oxidative stress via the activation of the SIRT1/Nrf2 and Keap1-mediated Nrf2/ARE signaling pathways (22, 23). In this cell model, H9C2 cells were made hypoxic using sodium dithionate in the ischemia buffer, and oxidative stress was induced by reoxygenating the cells with the normal media. The study first confirmed that LUA could effectively scavenges reactive oxygen species (ROS) in order to protect cardiomyocytes from oxidative damage induced by I/R. ROS can augment the release of proinflammatory cytokines and increase the expression of adhesion molecules that cause leucocyte accumulation, endothelial dysfunction, and myocardial cell death (18, 24, 25). It is reported that hydroxyl groups (-OH) at the C-4’ position are capable of aggravating the anti-inflammatory effect of flavones while -OH at the C-3′ position attenuates the function (26, 27). LUA retained -OH at the C-4’ position while getting rid of -OH at the C-3′ position in contrast with diosmetin, which partly explained the improvement in cell viability after I/R.

Calcium overload is involved in the pathological process of myocardial I/R injury. Sarcoplasmic reticulum Ca^2+^ ATPase (SERCA2a), a protein in charge of transporting calcium from the cytosol into the sarcoplasmic reticulum (SR) of cardiomyocytes, is essential in maintaining intracellular calcium homeostasis. Several reports indicated that SERCA2a expression and phosphorylation were down-regulated in myocardial I/R injury, leading to decreased calcium transporting capacity and ultimately apoptosis or necrosis of cardiomyocytes (28, 29). According to recent research, oxidative stress and calcium overload may work in concert to cause cell apoptosis. Zhu (30) found that mitochondrial calcium accumulation contributed to ROS generation and opening of the mitochondrial permeability transition pore (mPTP) to activate the caspase-9-dependent apoptosis pathway. Ma (31) showed that hypoxia/reoxygenation raised xanthine oxidase (XO)-dependent ROS overproduction, which in turn induced SERCA2 oxidation and inactivation, resulting in calcium overload and apoptosis in endothelial cells. Previously, luteolin pretreatment has been proven to enhance SERCA2a expression by up-regulation of the Sp1 transcription factor to reduce myocardial ischemia/reperfusion injury (5). Similar to luteolin, we found that LUA pretreatment ameliorated the intracellular calcium concentration induced by myocardial I/R, demonstrating that LUA might protect cardiomyocytes from I/R-induced apoptosis by suppressing calcium overload.

Mitogen-activated protein kinase (MAPK) signal pathway has a pivotal role in the regulation of the myocardial I/R process. MAPK is composed of JNK, p38, and ERK with different functions. JNK and p38 are involved in myocardial apoptosis, whereas ERK activation is necessary for cell survival and the recovery of ischemia-damaged myocardium (32). Evidence also indicated that the p38 MAPK pathway was activated during the I/R procedure while inhibiting it by SB203580 enhanced SERCA2α activity, relieved calcium overload, and thus reduced cardiomyocyte apoptosis (29). Luteolin was revealed to protect against myocardial I/R injury by inhibiting ROS-activated p38 and JNK pathways (29, 33). Accordingly, we found that LUA thwarted the phosphorylation of JNK and p38/MAPK evoked by I/R, indicating LUA might mitigate I/R injury in a JNK and p38/MAPK-dependent manner.

## Conclusion

In summary, the present study demonstrated that a newly synthesized flavone from luteolin named LUA significantly inhibited I/R-induced cardiomyocyte apoptosis, oxidative stress, and calcium overload by inhibiting JNK and p38/MAPK signaling pathways. In addition, LUA was probably to exert better effects compared to luteolin if administrated *in vivo* because LUA can avoid COMT-catalyzed methylation while luteolin cannot (Figure 9). These results potentially provide new insights and an experimental basis to promote the development of luteolin and lay a foundation for future applications of luteolin derivative in myocardial I/R injury. However, animal experiments are further needed to clarify the pharmacokinetics and protective effects of LUA on cardiovascular diseases.

## Authors’ Contributions

Z S and D H designed the experiments; Y L and X Y performed experiments and collected data; Y L, X Y, and Y L discussed the results and strategy; Z S and D H supervised, directed, and managed the study; Z S, Y Lin, and Y Li funded the study; Y L and X Y drafted the manuscript; Z S approved the final version to be published.

## Conflicts of Interest

It has been declared that the authors do not have any financial conflicts of interest.
